# Left Atrial scar assessment using imaging with isotropic spatial resolution and compressed sensing

**DOI:** 10.1186/1532-429X-14-S1-O8

**Published:** 2012-02-01

**Authors:** Mehmet Akcakaya, Susie Hong, Raymond H Chan, Tamer A Basha, Mehdi H Moghari, Kraig V Kissinger, Beth Goddu, Mark E Josephson, Warren J Manning, Reza Nezafat

**Affiliations:** 1 Medicine, Beth Israel Deaconess Medical Center, Harvard Medical School, Boston, MA, USA; 2 Radiology, Beth Israel Deaconess Medical Center, Harvard Medical School, Boston, MA, USA

## Summary

We assess left atrial scar using late gadolinium enhancement (LGE) with isotropic spatial resolution of 1.4mm^3^ by using highly accelerated LOST [1] reconstruction.

## Background

Atrial fibrillation (AF) is the most common sustained cardiac arrhythmia [2]. Pulmonary vein isolation (PVI) using radiofrequency (RF)-ablation is the leading treatment for AF. Recently, LGE imaging of the LA has been used to identify atrial wall scar due to RF-ablation [3]. However, current LGE methods have limited spatial resolution that substantially impact assessment of scar in the complex geometry of PVs and LA. In this study, we sought to utilize prospective random k-space sampling and LOST [1] for accelerated LGE imaging, where reduction in imaging time was traded-off for improved isotropic spatial-resolution.

## Methods

23 patients with history of AF (6 females, 58.1±9.6 years, 9 pre-PVI, 2 with history of PVI; 8 post-PVI; 3 with both pre and post-PVI) were recruited for this study. LGE images were acquired 10-to-20 minutes after bolus infusion of 0.2 mmol/kg Gd-DTPA. Free-breathing ECG-triggered navigator-gated inversion-recovery GRE sequences were used for all acquisitions (TR/TE/α=5.2/2.6ms/25°, FOV=320×320×100mm^3^). The PV inflow artifact reduction technique in [4] was also utilized. For each patient, a standard non-isotropic 3D LGE scan (1.4×1.4×4.0mm^3^) and a 3-fold-accelerated high-resolution 3D LGE scan (1.4^3^ mm^3^) were performed, with randomized acquisition order. For random undersampling, central k-space (45×35 in ky-kz) was fully-sampled, edges randomly discarded, and phase reordering performed as in [5]. Acquisition times were ~4 mins assuming 100% scan-efficiency at 70bpm for both scans.

All undersampled data were reconstructed offline using LOST [1]. LOST-reconstructed high-resolution, and standard LGE images were scored by two blinded readers for diagnostic value, presence of LGE(yes/no); and image quality in axial(Ax), coronal(Co) and sagittal(Sa) views (1=poor,4=excellent).

## Results

**Figure 1:**
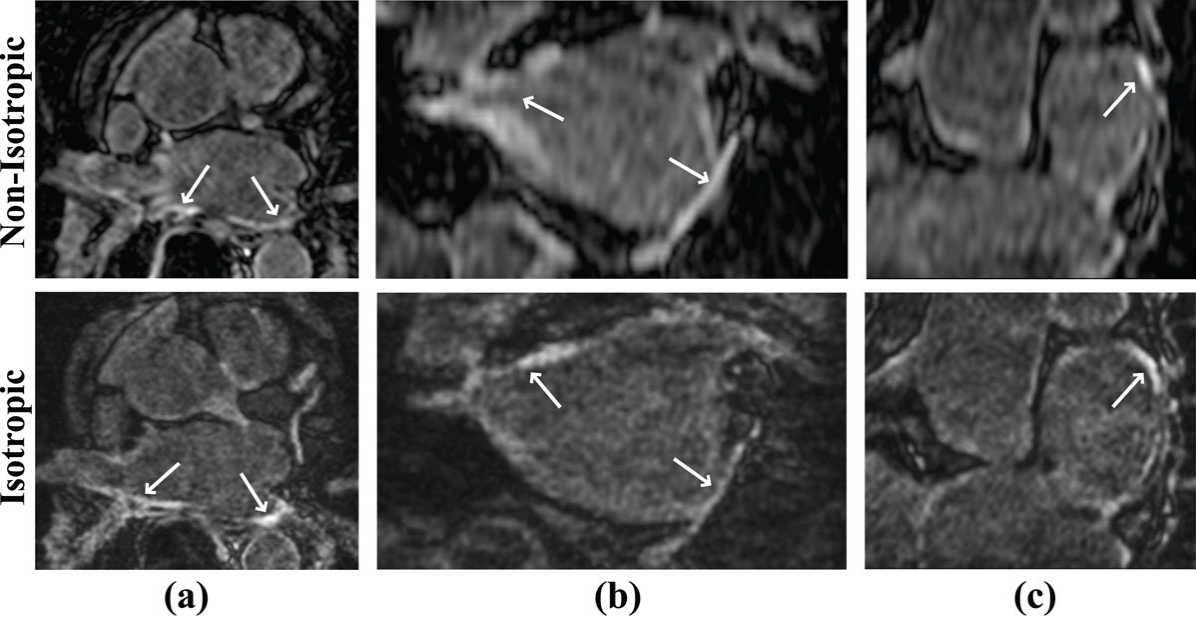
Axial (a), coronal (b) and reformatted (c) LA LGE images from a patient who underwent PVI. LOST-reconstructed images from the isotropic resolution scan with acceleration rate 3 enable better visualization from different orientations, whereas the standard non-isotropic LGE images suffer from blurring in sagittal and coronal views.

**Figure 2:**
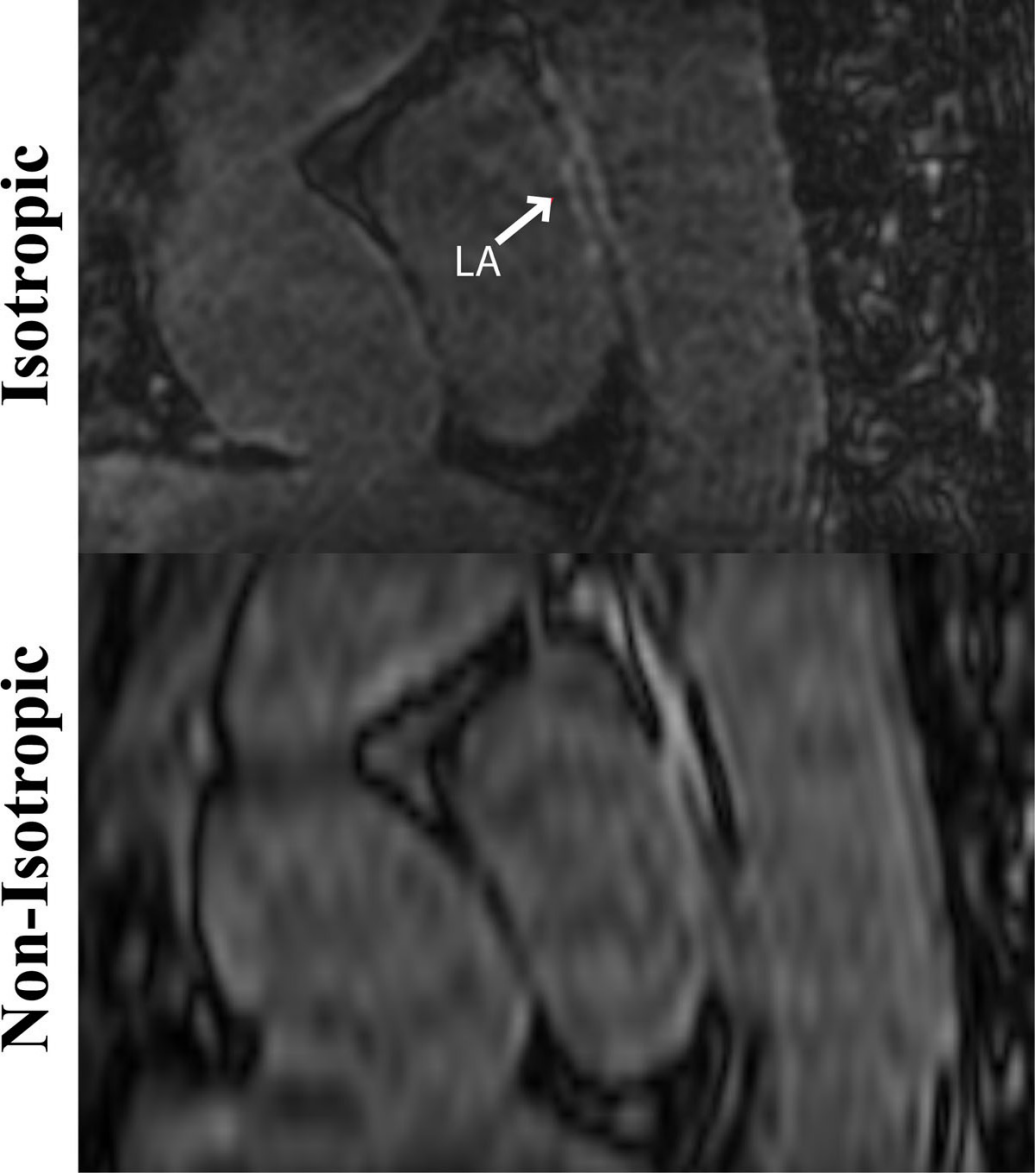
A sagittal view showing enhancement of the LA and the descending aorta in a patient who underwent PVI. The isotropic resolution of the LOST-reconstructed images from the accelerated scan allows clear visualization of separate regions of enhancement, whereas the non-isotropic resolution exhibits blurring of these regions.

Three cases were declared non-diagnostic due to contrast-washout and imperfect inversion-time. LGE was visually present in 14 of the remaining 20 patients based on standard-LGE images, and 12 based on LOST-reconstructed ones (disagreement on one pre- and one post-PVI patient). Figure 1 and 2 show comparisons of isotropic vs. non-isotropic LGE images in two patients. Image scores for LOST-LGE: Ax=2.90±0.70, Sa=3.33±0.66, Co=3.00±0.63; and standard LGE: Ax=3.76±0.54, Sa=2.48±0.60, Co=2.24±0.44, where differences were significant in all views.

## Conclusions

LOST allows isotropic spatial-resolution in LGE for assessment of LA and PV scar. Isotropic resolution allows reformatting LGE images in any orientation and facilitates assessment of scar. Further clinical study is needed to assess if the improved spatial resolution will impact the diagnostic interpretation of data.

## Funding

NIH R01EB008743-01A2.
